# 3D Modeling of Epithelial Tumors—The Synergy between Materials Engineering, 3D Bioprinting, High-Content Imaging, and Nanotechnology

**DOI:** 10.3390/ijms22126225

**Published:** 2021-06-09

**Authors:** Poonam Trivedi, Rui Liu, Hongjie Bi, Chunlin Xu, Jessica M. Rosenholm, Malin Åkerfelt

**Affiliations:** 1 Laboratory of Natural Materials Technology, Faculty of Science and Engineering, Åbo Akademi University, 20500 Turku, Finland; ptrivedi@abo.fi (P.T.); rui.liu@abo.fi (R.L.); Hongjie.Bi@abo.fi (H.B.); chunlin.xu@abo.fi (C.X.); 2 Key Laboratory of Bio-Based Material Science and Technology (Ministry of Education), Material Science and Engineering College, Northeast Forestry University, Harbin 150040, China; 3 Pharmaceutical Sciences Laboratory, Faculty of Science and Engineering, Åbo Akademi University, 20500 Turku, Finland; jerosenh@abo.fi; 4 Cell Biology, Faculty of Science and Engineering, Åbo Akademi University, 20500 Turku, Finland; 5 Institute of Biomedicine, University of Turku, 20520 Turku, Finland

**Keywords:** spheroid, organoid, tumor microenvironment (TME), extracellular matrix (ECM), biomaterials, 3D bioprinting, nanoparticles (NPs), high-content screening

## Abstract

The current statistics on cancer show that 90% of all human cancers originate from epithelial cells. Breast and prostate cancer are examples of common tumors of epithelial origin that would benefit from improved drug treatment strategies. About 90% of preclinically approved drugs fail in clinical trials, partially due to the use of too simplified in vitro models and a lack of mimicking the tumor microenvironment in drug efficacy testing. This review focuses on the origin and mechanism of epithelial cancers, followed by experimental models designed to recapitulate the epithelial cancer structure and microenvironment, such as 2D and 3D cell culture models and animal models. A specific focus is put on novel technologies for cell culture of spheroids, organoids, and 3D-printed tissue-like models utilizing biomaterials of natural or synthetic origins. Further emphasis is laid on high-content imaging technologies that are used in the field to visualize in vitro models and their morphology. The associated technological advancements and challenges are also discussed. Finally, the review gives an insight into the potential of exploiting nanotechnological approaches in epithelial cancer research both as tools in tumor modeling and how they can be utilized for the development of nanotherapeutics.

## 1. Introduction

Cancer is the second leading cause of mortality worldwide, and 90% of cancers are of epithelial origin, known as carcinoma. Carcinoma is a malignancy in the epithelial cells, which have a multidisciplinary role in protection, absorption, secretion, excretion, filtration, diffusion, and sensory reception in tissues. The World Health Organization (WHO) conducted a worldwide study in 2018, which showed that in both sexes, the leading cause of cancer deaths is lung cancer (18.4%). In males, prostate, colorectal, and liver cancers show the highest incidence after lung cancer, and stomach cancer shows the highest mortality, whereas in females, breast cancer is the leading cause of cancer deaths, followed by colorectal and lung cancer for incidence [1].

**Figure 1:**
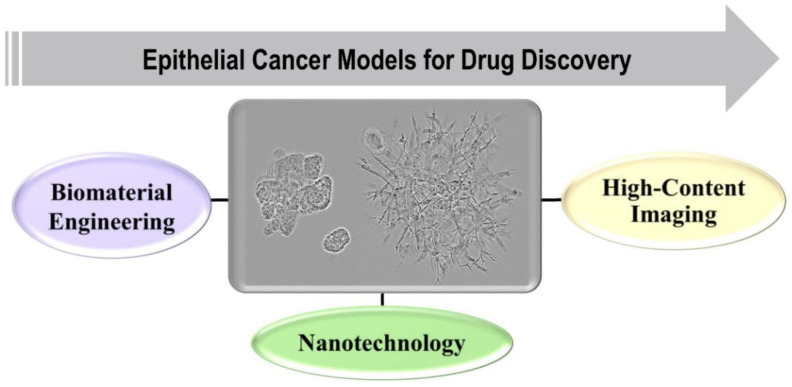
Research areas that are related to epithelial cancer and where 3D cell culture models are useful.

To study the origin, progression, metastasis, and underlying mechanisms of epithelial cancers, various models have been designed, utilizing multidisciplinary fields of science such as biomaterials engineering, nanotechnology, and high-content imaging. They have provided substantial information to improve anti-cancer drug discovery and diagnostics (Figure 1). The most simplistic model of in vitro cancer research is a two-dimensional (2D) monolayer culture of cancer cell lines, which, in spite of being the most utilized model, cannot provide realistic data about the heterogeneous, multicellular tumor microenvironment (TME) in the body. Currently, various models such as animals [2,3], transwells [4], spheroids [5,6,7,8], organoids [8,9,10], and xenografts [11,12] are utilized in cancer research with many advantages and disadvantages [13,14]. Hence, there is a need to develop models that can be utilized for monitoring cell growth, viability, polarization, and differentiation, as well as for and studying migration and invasion of tumor cells into the surrounding TME. Hence, a lot of attention has been focused on developing 3D model systems containing the extracellular matrix (ECM) of natural, synthetic, or semisynthetic origins using 3D bioprinting technologies that can provide more accurate information about the TME and cancer progression [14,15,16,17,18]. The 3D models can also serve as better choices for high-content screening approaches in the preclinical phase of drug development.

Anti-cancer drug development data show that ~5.1% of drugs that succeed to enter clinical trials eventually obtain approval from the Food and Drug Administration [19], and the cost of bringing a new drug to the market is over USD 2.6 billion [20]. A thorough understanding of TME, and occurrence mechanisms possess grand challenges in anti-cancer drug development research. The new era of anti-cancer drug development has been focusing on nanotechnological approaches for drug discovery research and diagnostic studies [21]. These can be utilized for the development of novel anti-cancer treatments, early detection of tumors, and discovery of cancer biomarkers [22]. The current scenario provides an opportunity to stimulate the preclinical testing phase by developing physiologically relevant models, which could reflect human tumor conditions using advanced imaging technologies for monitoring disease progression that also have the capacity of high-throughput screening in cancer research.

The review encompasses an overview of the crucial parameters to develop in vitro 3D models of epithelial cancers, especially breast and prostate cancer; followed by the current in vitro 3D experimental models in cancer research such as spheroid and organoid cultures, organ on chip models, ex vivo tissue slices, and applications of 3D culture in drug development. Further, 3D bioprinting technologies, associated challenges, and biomaterials used for organotypic 3D cancer models are discussed. Finally, insight is provided on how nanoparticles are being used in epithelial cancer research and how they can be utilized for high-content imaging approaches in drug discovery.

## 2. Key Parameters for Development of In Vitro 3D Models of Breast and Prostate Cancer

In cancer, the TME is heterogeneous in nature and is mainly composed of ECM and a stroma that includes fibroblasts, adipocytes, endothelial cells, immune cells, numerous growth factors, and cytokines [23,24]. ECM plays a crucial role as it acts as a reservoir of growth factors required for cell survival and normal tissue functions [25]. The ECM microarchitecture, composition, stiffness, and topography play a vital role in tumor progression and directly affect cell behavior [26,27]. It has already been shown that 3D in vitro models, which mimic the TME, can provide real insight into anti-cancer therapeutics and how they affect the tumor formation, progression, and associated molecular mechanisms in cancer treatment [28].

### 2.1. A Brief Summary of Breast Cancer

Breast cancer, being the most common cancer in the female population globally, has a few alarming characteristics, such as hypoxic environment, variance amongst patient-to-patient, and heterogeneity between cells within the tumor [29,30,31], and a potential to metastasize to lymph nodes and other organs such as bone, liver, lungs [32]. In order to understand the breast cancer origin, progression, and microenvironment that is extremely complex in nature, various approaches such as in vivo models, ex vivo models, and in vitro models have been applied to recapitulate at least the basic components that play a role in tumor progression [13,33].

In a normal functional mammary gland, the epithelium is composed of tightly attached epithelial and myoepithelial cells via cell adhesion molecules, resulting in hollow tubular structures [34,35]. In breast tumors the cell–cell adhesion decreases, resulting in fast dissociation from each other and the epithelium, rapid proliferation, and formation of a solid tumor in the lumen of epithelial cells, also known as ductal carcinoma [36]. Similarly, since the ECM is very much tissue- and location-specific, the normal mammary gland basement membrane is composed of entactin, collagen IV, and laminin proteins, which are involved in cell polarization, formation, and maintenance of acini (mammary gland lobules). While the normal ECM is composed of collagen I, lipids, and proteoglycans such as perlecan and tenascin [37,38], the biochemical composition of ECMs in the TME are different from the normal ones and result in different cellular behaviors [35]. The breast tumor tissues are also comparatively stiffer than normal mammary tissues, resulting in enhanced migration speed of the tumor cells, thus, promoting angiogenesis within the tumor [39,40]. The TME is also associated with a disorganized vascular network. The fiber structure, porosity, pore size collectively termed as microarchitecture, and signaling molecules such as cytokines, also play a significant role in tumor progression and invasiveness [41,42].

### 2.2. A Brief Summary of Prostate Cancer

Similarly, prostate cancer is the most common cancer diagnosed worldwide in men, especially in the western world. The prostate gland, which is phenotypically fibromuscular in nature with a canalized ductal-acinar structure, has tall columnar secretory luminal cells and a flattened basal epithelium that develops from the embryonic urogenital sinus [43]. Stem cells are the source of both prostate luminal and basal epithelia [44]. In research with advancing tools and technologies, various hypotheses undergo reformulation. For example, earlier, only luminal cells have been considered the cellular origin of most prostate cancers. However, recent research suggests that prostate basal stem and progenitor cells can also give rise to prostate cancer [45,46]. The present knowledge of the detailed prostate cancer-driving mechanisms is incomplete, and a deeper understanding is needed to predict tumor aggressiveness, efficient and sensitive modes of tumor detection [47].

The past and the ongoing research efforts in the fields of biomaterial and tissue engineering have provided a platform to design human representative 3D in vitro models mimicking human pathophysiological conditions. The 3D models allow, e.g., epithelial morphogenesis, including the formation of tubules and acini that are fully functional [48]. It has been shown that 3D in vitro models, which can mimic the TME and surrounding ECM’s biochemical composition, stiffness, micro-organization, and vasculature, can serve as representative model systems of breast and prostate cancer tumor research [35,49].

## 3. Current In Vitro 3D Experimental Models in Cancer Research

**Figure 2:**
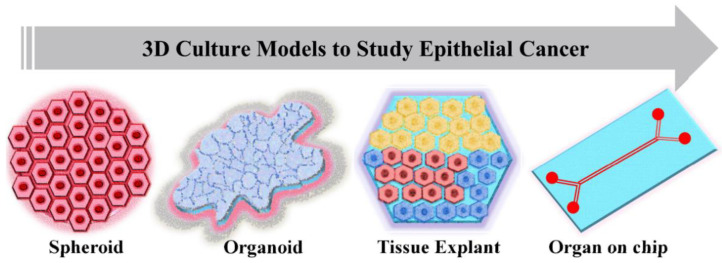
Current 3D culture models used in epithelial cancer research.

In vitro cancer models are the simplified versions in comparison to in vivo models when studying cancer mechanisms and the effect of anti-cancer moieties on tumor growth and progression. Standard 2D cell culture models fail to recapitulate the cellular mechanisms involved in tumor progressions, such as cell–cell adhesion, polarization, epithelial differentiation, mechanotransduction, invasion, and proper signaling of cells within the tumor tissues. Recent developments have shown that 3D in vitro models have tremendous potential in cancer research due to their most promising characteristic of very closely mimicking the in vivo model systems (Figure 2). An ideal in vitro tumor model should be able to recapitulate the 3D in vivo environment along with reproducing the interaction between tumor and stromal cells, thus regulating the cellular functions. Depending upon the method of cell seeding, the 3D in vitro models could be categorized as scaffold-based and scaffold-free models. The scaffold-based models utilize the prefabricated ECMs prepared from different materials such as natural or synthetic materials or decellularized ECM. In scaffold-free models, cells proliferate as non-adherent floaters without any support material, and 3D constructs are formed due to cellular self-assembly [50].

Based upon the cell culture methodology, the 3D models in cancer research can be classified into (i) non-adherent cultures as multicellular aggregates, (ii) culture on inserts, and (iii) embedded in natural or synthetic ECM. Here we discuss the most common modes of 3D cancer cell models.

### 3.1. Spheroid and Organoid Cultures

The most used 3D tumor models are spheroids and organoids: 3D spheroids are in vitro models which are cellular aggregates. Spheroids can be homotypic or heterotypic. The homotypic spheroids are composed of only cancer cells, while heterotypic are cultured with other cell types such as fibroblasts or immune cells [51]. They have been used to design models of various cancer types such as breast [51], prostate [52], and lung cancers [53]. Some of the most studied techniques for culturing spheroids and organoids in cancer research are [54,55]:

1. Cells cultured in suspension-based hydrogels in spinning flasks resulting in the formation of cellular aggregates with diverse morphologies.
2. Cells cultured in suspension condition using liquid overlay techniques in which the interaction between cells leads to the formation of non-adherent 3D cell aggregates.
3. Hanging drop techniques in which non-adherent spheroids are formed in cellular droplets.
4. Tapping chamber of the microfluidic reactor where cells, after injection, fuse and form non-adherent spheroids in a controlled manner.
5. Cells embedded in ECM in multi-well plates, where either round spheroids or invasive tumoroids are generated, and the cells adhere to and interact with the surrounding ECM.

The internal structure of non-adherent 3D spheroids can, to some degree, mimic the solid tumor architecture, and it is comprised of different cell layers. The core is composed of necrotic cells, while the middle layer has mostly senescent cells. The necrotic or senescent cells of the inner layer are dedicated to the absence or deprivation of nutrients and hypoxic environment, which results in the accumulation of lactate in the spheroids, same as that of in vivo solid tumors. The outer layer is formed of cells with high proliferating rates due to convenient access to oxygen and nutrients [56,57]. The therapeutic efficacy of anti-cancer drugs such as cisplatin, doxorubicin, which promote cancer cell death through the formation of reactive oxygen species and drugs, is hampered due to the layered organization in spheroids [58].

In contrast to non-adherent cultures, tumor cells can also be cultured embedded in ECM, where they spontaneously form 3D structures of organotypic nature, which can be called spheroids if they are round or tumoroids if they have an invasive appearance. Here, single cells that are embedded into ECM grow into multicellular structures of organotypic nature. Each of the functional structures is of clonal nature but often has characteristic phenotypes that correspond to different tumor stages. Normal epithelial cells or non-aggressive cancer cells can form well-differentiated, polarized, round spheroids with functional basement membranes. In contrast, tumoroids formed by aggressive cells mainly result in undifferentiated clusters of cells or massive invasive structures. In epithelial cancers like breast and prostate, invasion through the ECM is typically of the collective type, and typically invasion is less frequently observed [18,59]. Tumor cells can also be embedded together with stromal cells and be co-cultured in the ECM. Incorporation of stromal cells such as CAFs will promote genuine, functional interactions between the different cell types, which can be observed in vivo [60]. 3D organotypic cell cultures can therefore act as a bridge between traditional 2D cell culture and costly animal models.

In comparison to spheroids, organoids are more advanced 3D in vitro multicellular structures that mimic the corresponding architecture of in vivo organs. The term organoid is mostly used to describe structures obtained in 3D culture derived from stem cells that are isolated from primary patient samples. The complexity of an organoid is regulated by the developmental potential of the starting stem cells [61]. The organoids can, like the spheroids, be cultured in non-adherent conditions or embedded in ECM. Organoids are mostly used for translational epithelial research, patient-specific treatment planning, and disease modeling due to their close resemblance to the native tissue composition. However, the 3D organoid culture is advantageous over 3D spheroids due to enhanced physiological and clinical functions. The 3D organoid models of various tumor types have provided concrete evidence to validate the use of these models [62,63,64,65,66]. The patient-derived tumor organoids are known to accurately resemble the molecular, genetic, morphological, functional, and architectural pathophysiological characteristics of cancer. Thus, in the future, with continuous development, they can provide substantial information in cancer research.

### 3.2. Organ on Chip Models

These are the more advanced versions of in vitro models in which chips of optically transparent materials such as plastic, glass, or PDMS elastomers are designed to have hollow microchannels for perfusion. The cells of choice are cultured in the micro-channels, and the cellular viability is maintained via perfusion with the culture medium through the endothelial cell-lined channels, which are supposed to mimic the structural and functional responses of the tissues and organs under in vitro environment [67]. To mimic the physical microenvironment of living organs, such as breathing motions in the lungs, mechanical forces can also be applied. The concept of a tumor on a chip has provided a novel strategy to design a controlled TME model for studying tumor growth, metastasis, and anti-cancer drug responses [67]. In a study to mimic the origin and progression of tumor nodules from normal breast epithelial tissue, a mammary duct model was developed [68]. The tumor on a chip model has also been tested to study nanoparticle transport in cancer cells [69].

### 3.3. Ex Vivo Tissue Slices

Ex vivo tissue slice models are based upon the concept of directly taking living tissues from a living organism and performing the experiment with minimalistic alterations in the organism’s natural conditions. In cancer research, the respective tumor tissues are sliced into thin sections and mounted onto porous membranes, and incubated under controlled conditions. The tumor tissue slices are considered to maintain the 3D structure with extra and intercellular interactions and retain metabolic capacity. In the first human tumor histoculture studies, collagen was used as a support matrix when growing tumors. The results showed that directly obtained tissue slices from surgery can grow at a high rate in vitro for a longer time maintaining many in vivo characteristics such as 3D growth, retention of differentiated function [70]. The ex vivo tissue slices can be cultured via submersion, grid, or sponge techniques. In the submersion technique, the tissue slices are completely submerged in culture media. While in the grid method, the tissue slice is kept in contact with the media through a matrix, supported by a metal or plastic grid. The sponge technique utilizes gelatin or collagen as a sponge to culture the tissue slices in culture media [71]. The gelatin sponge method has provided relevant information in the case of breast and prostate tumors [72,73]. These models are typically used in cancer drug discovery to study the tumor response to newly developed anti-cancer moieties [74]. Although these models have shown potential to assess the tumor intrinsic resistance or sensitivity to different anti-cancer agents, with time, there is a loss of integrity, thus prohibiting the use of these models to study drug resistance or metastasis mechanisms.

### 3.4. Applications of 3D Cultures in Drug Development

There is increasing evidence that 3D cell culture models can more accurately predict therapeutic efficacy in comparison to standard 2D cell cultures [75,76]. For example, certain cancer cell types cultured in 2D may be more sensitive to the toxic effects of drugs than when cultured in 3D, where they form larger structures [77]. For some cell types, it has been observed that a hypoxic TME, which can be generated in 3D spheroids, also can lead to a decrease in drug sensitivity [6]. A practical example study is breast cancer MCF7 cells cultured in 3D, which demonstrated a significantly stronger resistance to tamoxifen compared to 2D cultures of the same cells [78]. Another example provided evidence for strong changes in proliferation and metabolic capacity of colon cancer spheroids in 3D compared to 2D. These 3D models displayed increased anti-tumor responses to AKT-mTOR or MAPK-pathway inhibition compared to those in 2D models [79]. These and many more studies have shown increased biological relevance of 3D cultures over 2D cultures in terms of drug responsiveness at dosages that mimic the in vivo responses. Additionally, 3D cell culture enables co-cultures of cancer cells and stromal cells in tissue-like in vitro models to mimic, e.g., fibroblast-tumor crosstalk or immune responses to cancer [60,80]. Currently, the pharmaceutical drug development process relies heavily on the use of animal models for preclinical drug sensitivity tests. However, recent comparisons of drug toxicity between animals and humans have challenged the presumption that animal models most reliably predict the sensitivity of novel drug treatments [81]. Additionally, 3D cell models could also be used to bridge the gap between simplified 2D cell cultures in vivo studies in animals. The incorporation of human cells and tissues in 3D platforms used in drug development has the potential to significantly improve the predictive value of drug sensitivity testing.

## 4. 3D Bioprinting Technologies and Associated Challenges

Three-dimensional bioprinting is a technology in which 3D structures are fabricated by layer-by-layer precise deposition of biological materials, living cells, and biochemicals. In cancer research, 3D bioprinting technology has provided hope and motivation to design models to recapitulate the in vivo TME to study cancer genesis, mechanisms, and to facilitate drug development screening by conveniently combining patient-derived cells and materials [82]. Ideal material for 3D bioprinting must meet the most important criteria, such as easy handling and deposition by the bioprinter, biocompatibility, structural and mechanical stability, tissue-specific material biomimicry, and minimalistic nontoxic byproduct generation. Herein, we discuss the most studied 3D bioprinting technologies and associated challenges.

### 4.1. Bioprinting: Types of 3D Printing Technologies

**Figure 3:**
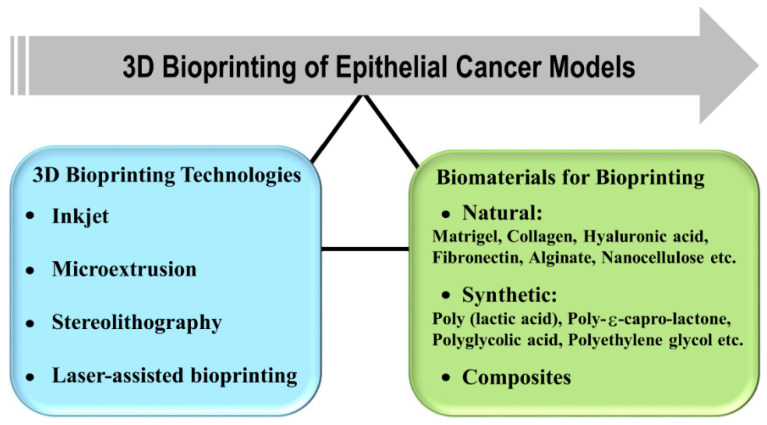
3D bioprinting technologies and classification of biomaterials in bioprinting to design epithelial cancer models.

In 3D bioprinting, the most important factors to be considered are biological materials used for printing, cell viability, and surface resolution. The current 3D bioprinting technologies are inkjet-based [83], microextrusion [84], stereolithography (SL)-based [85], and laser-assisted printing [86] (Figure 3). The inkjet-based 3D bioprinters were originally modified versions of 2D ink-based printers in which the ink is replaced by biological material. It works on the principle of generating bioink droplets at the printhead with the energy provided either by a heater or a piezoelectric actuator. The major common limitation of inkjet bioprinting is that the biological sample has to be in a liquid state to enable droplet formation followed by self-solidification to form organized 3D structures. Various groups have tried to address the limitation by utilizing cross-linking strategies such as chemical or UV light exposure after droplet formation. However, the cross-linking process may slow down the overall bioprinting process, affect the natural composition of extracellular material, and could also be toxic to the cells [87,88]. Compared to other technologies, the inkjet process also offers advantages such as high speed, high resolution, simplicity of operation, low cost, and compatibility with numerous biological samples.

In microextrusion 3D bioprinting, the robotically controlled printers extrude a continuous stream of bioink through a nozzle utilizing mechanical or pneumatic forces, thus, resulting in layer-by-layer deposition onto a substrate by a microextrusion head [89,90]. This technique is often described as direct ink writing (DIW), which may be equipped with UV-led sources to solidify the printed scaffold substrate in situ [91]. The substrate can be a culture dish (solid), growth medium (liquid), or material derived from the gel. The final bioprinted structure characteristics are directly dependent upon various parameters such as nozzle diameter, extrusion pressure, speed, temperature, UV-led curing, and many more. The rheological properties such as viscosity, shear thinning of the polymer or hydrogel employed play a vital role in designing the table 3D construct. It has been shown that the materials showing shear thinning behavior are preferred in microextrusion applications. During biofabrication, the high shear rate at the nozzle tip facilitates the material flow, and upon deposition, the viscosity of the material decreases automatically with a decreased shear rate. The technique has been utilized to fabricate various tissue and tumor models [92,93,94].

SL-based printing mainly includes projection-based digital light processing (DLP) and laser-based stereolithography apparatus (SLA), due to its high printing precision, excellent surface quality, and defect-free printing process has received widespread attention [95,96]. SL-based 3D printing applies light exposure (usually UV light) to convert photosensitive materials into cured solids in a layer-by-layer fashion [95,96]. The photosensitive material can be customized and formulated to have a single component or multiple components, such as biological macromolecules [96], multifunctional nanocomposites [97], and even living cells [85], which can simultaneously integrate the scaffolds’ bioactivity and function for various biomedical applications. SL printing technology offers a universal 3D printing platform for tissue engineering with high precision.

Laser-assisted bioprinting (LAB) is another promising 3D bioprinting technology that has displayed successful compatibility with biological molecules such as DNA, as well as cells [98,99]. It has shown the potential to print mammalian cells with a minor negative impact on cellular viability and functions [100]. The technology uses focused laser pulses on the absorbing layer of the ribbon to generate a high-pressure bubble that propels cell-containing materials toward the collector substrate. The LAB resolution performance is affected by the following factors: surface tension, wettability of the substrate, thickness, and viscosity of the biological material layer [101]. Until now, LAB has been explored in the area of mostly tissue engineering, but there are hopes of utilizing the technology to also explore the potential to design tumor models in the future.

### 4.2. Challenges Associated with 3D Bioprinting Technology

Technological advancements are always accompanied by associated challenges, so as in 3D bioprinting. Since 3D bioprinting involves interdisciplinary aspects of science, from technology to biomaterials, the associated challenges also encompass these areas. The technical issues comprise the need for high resolution, increased speed, and acceptable compatibility with biomaterials. The ultimate aim of 3D bioprinting is to design structures recapitulating specific human tissues or organ structures. The complexity of biological responses and gradients of ECM in tissues makes it even more challenging to construct 3D-bioprinted tissue-mimicking structures. In the current scenario, the choice of materials used for bioprinting is based upon their compatibility with the cell function and growth, or better extrusion performance, or better-cross-linking properties to provide a stable structure after bioprinting. The optimal material should also meet the requirements of TME [102,103]. Some of the materials used are collagen, hyaluronic acid, alginate, photocurable acrylates, and many more. For instance, a new group of plant-derived biomaterials, alginate and wood nanocellulose, are found to be promising for scaffold construction [91,104], even though in vivo biodegradability remains a concern. The range of compatible materials, deposition methods, and cells can be extended in the future with better understanding and advancement in the preexisting 3D bioprinting technologies.

## 5. Biomaterials for Organotypic 3D Cancer Models

The complexity and heterogeneity of tumors present the biggest challenge in modeling the tumor and tumor microenvironment. Biomaterials can be used to create defined macro- and microenvironments, which have the potential to manipulate cells and tissues in vitro and in vivo. In the early 1980s, the concept to utilize biomaterials to study tumor biology was attempted to know how and whether the signals from the extracellular material regulate cellular behavior. The biomaterials based upon origin can be broadly classified into natural, synthetic, and hybrid materials (Figure 3). The natural biomaterials can be further classified into animal and not animal-based. The most used ECM-derived biomaterials in cancer research are collagen, laminin, hyaluronic acid, and reconstituted basement membrane or Matrigel^®^. These biomaterials have promising characteristics such as cytocompatibility, the ability to be remodeled by cells along with intrinsic cell adhesion properties. Still, there are associated challenges to study the influence of ECM on tumor cells due to uncontrolled degradation of natural biomaterials, batch to batch variability, and complex molecular composition [105,106,107]. Synthetic biomaterials can, therefore, provide more precise control over biochemical and mechanical properties when modeling the ECM of tumors. However, as the synthetic biomaterials lack natural cell adhesion sites, they are not remodeled by cells [108]. In this section, we have discussed the most studied biomaterials in epithelial cancer research and current progress in the area.

### 5.1. Animal Based Biomaterials

#### 5.1.1. Matrigel

The first successful biomaterial used to study tumor biology was derived from Engelbreth-Holm-Swarm (EHS) mouse sarcoma cells, also known by the tradename Matrigel [109]. Matrigel is mainly composed of assorted ECM proteins such as laminin, type IV collagen, heparin sulfate proteoglycans, and entactin, along with growth factors. It is mostly used to support the formation of epithelial structures by partly mimicking the in vivo cellular microenvironment thus, recreating in vitro biochemical and architecture, such as the basement membrane. It has been very widely used for studies of both in vitro and in vivo tumor growth and morphologies of various cancer cell lines and tumor types [110,111]. It has also been used to study 3D co-cultures of tumor cells grown together with stromal cells, e.g., fibroblasts, natural killer cells and T lymphocytes [60,66,112], investigation of cancer cell migration and invasion [113], morphological changes [18,59,114], and differentiation processes [115].

#### 5.1.2. Collagen

Collagen I is the most abundant ECM protein in tumor stroma. Its major role is to provide structural integrity and mechanical support to the tissues. The motifs in the collagen fibers that allow cells to adhere and proliferate are composed of arginine-glycine-aspartic acid (RGD) amino acid sequences. Physically cross-linked 3D collagen hydrogels have been used to encapsulate prostate, breast, and lung cancer cells. The metastatic MDA-MB-231 breast cancer cells displayed tissue-thickness dependent hypoxia and central necrosis in collagen hydrogels as compared to standard 2D-cultured cells [116,117]. While non-metastatic MCF-7 breast cancer cells showed increased cancer stem cell signatures and epithelial-mesenchymal transition (EMT) markers when cultured in 3D collagen hydrogels, which indicates tumor heterogeneity and malignant progression [118]. Collagen hydrogels designed by enzymatic cross-linking using transglutaminase have also been studied to culture prostate, breast, and bone cancer cells [119]. In a study, the cancer cell migration was investigated utilizing the fibrillar nature of collagen hydrogels and the possibility of tuning various properties such as cross-linking density, pore size, elastic modulus, and fiber alignment. The increased stromal collagen density is generally an indication of malignant invasion owing to higher tissue stiffness. Studies performed with in vivo mouse models, in vitro mammary epithelial organoids, and self-aggregated tumor spheroids demonstrated that enhanced stromal collagen content and fibrillar architecture promote tumor formation and metastasis [120,121]. We can clearly conclude that the physiochemical properties of the collagen in hydrogels regulate the fate of the cancer cells.

#### 5.1.3. Hyaluronic Acid (HA)

Hyaluronic acid is a high molecular weight biomaterial present in various tissue ECM. It is mainly composed of repeating units of D-glucuronic acid and D-N-acetylglucosamine. Hyaluronic acid can be chemically modified with methacrylate groups or thiol groups, which can be transformed into robust hydrogels for tumor modeling and bioprinting of cellularized structures [122,123]. The molecular weight of the HA plays a crucial role in cancer progression and malignancy. It has been shown that prostate cancer cells cultured within HA hydrogels (molecular weight: 0.5−1.3 MDa) resulted in cluster-like formation with invadopodia suggesting 3D invasiveness through the activity of motility-associated pathways and expression of hyaluronidases enzyme [124]. The enzyme hyaluronan synthases (HAS1, HAS2, and HAS3) synthesize HA (low and high molecular weight) and thus controls the HA molecular weight, while hyaluronidase enzymes (HYAL1, HYAL2) are responsible for the degradation of high molecular weight HA into low molecular weight. It has been shown that the increased levels of low molecular weight HA along with increased expression of HAS2, HYAL1, and HYAL2 were responsible for breast cancer cell invasion and metastasis [125]. Chemically modified HA hydrogel with reactive thiol and acrylate groups was investigated with the LNCaP prostate cancer cells, which showed enhanced pro-angiogenic activity as compared to 2D cultured cells [126]. Thus, we can conclude that the macromolecular and biochemical characteristics of HA have a direct impact on designed hydrogels, which correspondingly regulate the cellular response and cell-matrix interactions in 3D cancer studies.

#### 5.1.4. Fibronectin

Fibronectin (FN) is a glycoprotein having three repeating units named type I, II, and III containing various binding sites for extracellular material components such as collagen/gelatin, fibronectin, heparin, growth factors, and others [127]. It has been studied that growth factor-fibronectin synergistic interactions have the potential to alter cell behavior, such as improved cell migration, proliferation, and differentiation. It has been shown that the fibronectin matrix deposited in TMEs promotes tumor progression but is contradictorily related to a better prognosis [128]. In the case of normal adult breast tissues, the ECM is mainly devoid of FN, whereas high FN levels have been detected in the stroma of breast tumors.

Since the role of FN in cancer is established, various FN-targeting approaches have been proposed to study their promising role in cancer imaging and therapy [129]. It has been proposed that the limitations of synthetic hydrogel matrices in cancer research, such as poor cell attachment, absence of growth factors in the solid phase, and lack of cell-mediated degradability can be addressed by designing FN-based hydrogels. These hydrogels can also serve as an alternative to natural extracellular material-derived matrices. In a recent study, full-length fibronectin-polyethylene glycol-based hydrogels of controlled stiffness were designed to enable the solid-phase presentation of growth factors in a physiological manner showing the potential to replace Matrigel [130].

### 5.2. Non-Animal Based Biomaterials

#### 5.2.1. Alginate

Alginate is the most abundant marine biopolymer with a polyanionic character and is extracted from various species of brown seaweed and bacteria, Pseudomonas, and Azotobacter [131]. It occurs in the form of calcium, sodium, and magnesium salts of alginic acid in the seaweed. Chemically it is composed of (1,4)-linked b-D-mannuronic (M block) and a-L-guluronic (G block) acids [132]. The ratio between the G and M blocks depends on the seaweed source from which it is extracted and the culture or growth conditions. The high content of guluronic blocks (G block) has been reported in alginates derived from seaweed stems, whereas alginates derived from seaweed leaves have shown higher contents of mannuronic blocks (M block). Alginates with higher contents of GG blocks are shown to form stronger gels than those with a high content of MM blocks due to their greater ability to bind calcium [133]. The G blocks are known to provide rigidity to the polymeric structure, and a higher concentration of G blocks in alginates results in the formation of stronger gels [134,135]. Due to the polyanionic nature, alginate forms complexes with positively charged species such as metal cations. It is believed that cations prefer to bind the G blocks of the chains, but recent studies also suggest that the M block also plays a crucial role in cross-linking the polymer chains [136]. The main difference at the molecular level between algal and bacterial alginates is the presence of O-acetyl groups at positions C2 and/or C3 in the bacterial alginates [137]. Alginate has been explored for cell encapsulation in many studies due to thermally stable gelation under cold conditions and ion exchange reactions [138]. Alginate hydrogels have been explored for the 3D encapsulation of breast cancer cells [139], human leukemia cells [140], and enrichment of cancer stem cells from human hepatocarcinoma cells [141,142]. Recently a hybrid hydrogel of Alginate and Matrigel was investigated with breast cancer cell lines MDA-MB-231 to design 3D material to be successfully used as a substrate for breast cancer cell culture [143].

#### 5.2.2. Nanocellulose

Cellulose is the most abundant biopolymer on earth and acts as the main structural support in plants. The para-crystalline microfibrils and nanofibrils have a major role to play in providing structural strength. Nanocellulose can be categorized into (1) bacterial nanocellulose (BNC), (2) cellulose nanofibrils (CNFs), and (3) cellulose nanocrystals (CNCs). BNCs are synthesized via a bottom-up approach where glucose is transformed into cellulose via an enzymatic process [144], while CNFs and CNCs are prepared via top-down approaches such as mechanical, chemical, or enzymatic treatment [145]. The major difference between CNFs and CNCs is that CNFs are composed of both crystalline and non-crystalline regions in the fibers, whereas CNCs have only crystalline regions. The presence of only crystalline regions in CNCs is attributed to the process of manufacturing, which involves hydrolysis. During hydrolysis, the non-crystalline regions are the first to be attacked by acid leaving behind the crystalline regions. Owning to the intrinsic characteristics, such as biocompatibility, non-cytotoxicity, tunable 3D architecture, and porous microstructures, and desired mechanical properties, nanocellulose is currently under intensive study in tissue engineering applications. The good biocompatibility of wood-derived nanocellulose has been verified with respect to crucial cellular processes involved in the growth and proliferation of fibroblasts [146]. Lou et al. successfully cultured human pluripotent stem cells in 3D nanocellulose hydrogels [147]. Nanocellulose hydrogel was formulated into 3D printing inks for fabricating 3D platforms with different cross-linking strategies for cell culture studies towards various medical application possibilities [148,149,150]. Recently a nanofibrillated cellulose-based hydrogel available by the trade name GrowDex® [147], with a successful commercialization breakthrough has been shown to mimic the ECM and support cell growth and differentiation with various cell lines. There are various other natural materials, such as chitosan [151] and agarose [152], which have shown the potential of mimicking ECM and supporting cell growth and differentiation.

### 5.3. Synthetic Polymers

#### 5.3.1. Poly (Lactic Acid)

Poly (lactic acid; PLA) is a product of the polyesterification reaction of lactic acid [153,154,155,156]. PLA has characteristics of non-toxicity, thermal stability, biocompatibility, and biodegradability. PLA is a thermoplastic polymer that is derived from renewable resources such as corn starch and sugar cane. There are many various types of PLA, including poly (L-lactic acid; PLLA), poly (D-lactic acid; PDLA), and poly (D, L-lactic acid; PDLLA), which result in a PLA group with a broad range of physiochemical properties [154,156]. PLA has been approved by the FDA as a hydrophobic aliphatic polyester in different biomedical and clinical applications as it degrades to the physiological product lactic acid [154,157,158]. The PLA could successfully be printed in a 3D scaffold, which provides sufficient mechanical integrity, biodegradability, and the metabolic activity and cell viability of bone mesenchymal stem cells (BMSCs) cultured on the scaffold were not affected [154]. The results of the detection of osteosarcoma cells showed that PLA scaffolds are non-cytotoxic and could promote cell growth, cell viability, and osteogenic gene expression [154].

#### 5.3.2. Poly-ɛ-Capro-Lactone

Poly-ɛ-capro-lactone (PCL) was widely used in biomedical fields due to its easily manipulable mechanical properties, biocompatibility, biodegradability, and non-toxicity, and was approved by the FDA as an implantable polymer material [154,159]. PCL is a saturated aliphatic polyester composed of hexanoate repeated units. It has a semicrystalline structure and a low glass transition temperature (−60 °C), giving it flexibility and softness at body temperature. The low melting point (about 60 °C) of PCL is advantageous for its use in extrusion-based 3D printing [155]. Due to their ability to be completely degraded by fungal and bacterial enzymes, PCL-based materials are of particular interest in the application of biodegradable materials. In addition, PCL-based formulations, whether as blend or copolymer with synthetic or other biopolymers, have attracted great interest in the applications in controlling drug delivery systems, cell cultures, and regenerative medicine implants as tissue engineering materials due to their remarkable permeability, non-toxicity, and excellent biocompatibility [160].

#### 5.3.3. Polyglycolic Acid

Polyglycolic acid (PGA) is a semicrystalline polymer with a high tensile modulus, which is insoluble in water and most organic solvents, but can be hydrolyzed in the presence of water, degraded in vivo to oligomers or glycolic acids, and eventually participates in the tricarboxylic acid cycle or is excreted in the urine [154,155]. PGA allows diffusion of nutrients upon implantation and subsequent neovascularization due to its high porosity [161]. In addition, PGA can be fabricated into different shapes and easily handled. PGA was the first synthetic polymer approved by the FDA for use in the production of absorbable sutures [162]. However, due to the rapid absorption of PGA, the degradation products such as glycolic acid and other acidic products can cause a strong inflammatory response limiting its application in biomedical applications. In order to adjust the degradation rate and mechanical property, PLA and PGA are often synthesized as the copolymer poly lactic-co-glycolic acid (PLGA). PLGA is a linear copolymer, which has great potential in biomedical applications due to its safety, good mechanical properties, good cell adhesion, and controllable degradation rate. PLGA has also been approved by the FDA for clinical use because it alleviates the shortcomings of the PLA and PGA. By changing the ratio of the two monomers of lactic acid and glycolic acid, the degradation rate of PLGA products can be tailored. Therefore, PLGA is preferred over PGA for use in a variety of biomedical applications, such as sutures and cancer drug delivery systems [156].

#### 5.3.4. Polyethylene Glycol

Polyethylene glycol (PEG) is a synthetic polymer widely used in the formation of multicellular tumor spheroids (MCTS) [163]. PEG is a versatile polymer that is resistant to protein adsorption, biocompatible, degradable, and hydrophilic, and PEG encourages cell-to-cell adhesion [164]. PEG has been largely used as a 3D support for tissue engineering, also because of its adjustable mechanical properties that allow easy regulation of the scaffold structure [164,165]. Covalently cross-linked PEG-based scaffolds can be synthesized by chain growth, step-growth, or mixing, and the cross-linking mode affects the number of structural defects and subsequent mechanical properties [166]. Compared with many natural biomaterials, PEG-based hydrogels have the advantages of easy adjustment, good stability, low cost, and good repeatability. PEG hydrogels are commonly used to form hepatocellular carcinoma MCTS because hepatogenic cells must be cultured in MCTS to maintain the liver-specific function, and PEG-based hydrogels maintain a high level of this function [165]. PEG-based hydrogels have also been used to form several other types of MCTS, including breast cancer and lung adenocarcinoma [157,162,163].

There are various other synthetic polymers for 3D culture such as poly (vinyl alcohol) (PVA) [162], poly hydroxyalkanoates (PHA), and their copolymers [167], which can be used in regenerative medicine for stem cell progenitor cell differentiation experiments.

### 5.4. Composites

The use of various materials in the biomedical field has recently experienced rapid growth. Single-class materials may not be able to satisfy all of the requirements for a given implant application. Therefore, many researchers combine two or more classes of composites with multiscale structures, and the desired properties for specific applications are thereby achievable, such as enhanced biocompatibility and biomechanical properties [153,168,169]. Some examples are given to reveal the applicability of biocomposites.

A new electrohydrodynamic jet 3D printing technology was developed by combining the effect of electrohydraulic force and thermal convection and verified the feasibility of preparing the controllable fiber composite scaffold. The method introduced an effective thermal field under the needle and improved the viscosity of the ink, the control of the injection morphology, and the solidification of the printing structure. Through theoretical analysis and experimental characterization, the formation mechanism of thermal convection on jet morphology and print structure characteristics was studied. Under the optimized conditions, a stable and finer jet was formed. Using this jet, various 3D structures can be printed directly under the condition of a large aspect ratio of ~30. Furthermore, the PCL/PVP composite scaffolds with the controllable filament diameter (~10 μm), which is close to living cells, were printed. Cell culture experiments showed that the printed scaffold had good cell biocompatibility and promoted cell proliferation in vitro. The development of electrohydraulic jet 3D printing technology provides a new way to directly print synthetic biopolymers into tissue engineering structures with a flexible scale [170].

A polylactic acid-hydroxyapatite (PLA-HAp) composite that can be processed by a 3D printer was demonstrated and developed. This material has proved to be a viable option for the development of implants for therapeutic bone regeneration. Biocompatibility in vitro was confirmed by cell viability studies, using osteoblasts MG63 cell lines, and the presence of polymer matrix HAp in enhanced cell attachment and osteogenic ability. This study lays the foundation for further research into the possibility and safety of 3D printing, polymer-based, absorbable composites for bone regeneration [153].

Wang et al. constructed a novel drug delivery system with targeted and controlled release capabilities [169]. The environmentally friendly polymer (CMC-G-PLA) obtained by grafting polylactic acid onto carboxymethyl cellulose biopolymer is amphiphilic and easy to form micelles, which can be used as a hydrophobic drug carrier. To achieve the effect of targeted therapy, anti-EpCAM antibodies were anchored to the polymer chain of carboxymethyl cellulose through amidation reactions. Adriamycin and HepG2 cells were used as model anti-tumor drugs and tumor-targeting cells, respectively. The core and shell structure, critical micelle concentration, drug loading rate, and encapsulation rate were studied by transmission electron microscopy, pyrene fluorescence probe, and UV standard curve. In addition, pH-induced release function test, cytotoxicity test, and in vivo tumor treatment test were carried out in mice. The results showed that the prepared antibody drug carrier could effectively deliver hydrophobic drugs to specific tumor sites and improve the therapeutic effect of the tumor. This versatile design provided new solutions for future cancer treatment [169].

Bishi et al. studied the liver trans-differentiation potential of human mesenchymal stem cells (MSC) on a biocomposite poly(l-lactic acid)-co-poly(ε-capro-lactone; PLACL) and prepared PLACL/collagen nanofibrous scaffolds [168]. PLACL is a highly hydrophobic polymer that is preferred for a variety of tissue engineering applications when mixed with collagen. Their results showed that PLACL/collagenous nanofibrous scaffolds are potentially biogenic and enhance the transformation and differentiation of human MSCs into functional hepatic globules under the induction of hepatogenic growth factors/cytokines. In another study, Shim et al. obtained PCL/PLGA/collagen scaffolds by a solid freeform fabrication method [171]. It was found that the activity and albumin secretion capacity of rat primary hepatocytes were mostly retained for more than 10 days in the PCL/PLGA/collagen scaffold but not in the PCL/PLGA scaffold. The results showed that the addition of collagen improved the cellular compatibility of the scaffold and promoted the proliferation and differentiation of hepatocytes [168].

## 6. A Niche for Nanoparticles in Epithelial Cancer Research

As exemplified above, by combining two or more types of multi-scale structural materials, e.g., natural ECMs and synthetic polymers, the resulting composite materials can achieve ideal properties for specific applications, such as biocompatibility and biomechanical properties. Among the composite scaffold designs, attention has also been drawn to material groups of polymers and nanoparticles, taking advantage of each individual material to promote their medical applications. During the past two decades, nanotechnology has been rapidly advancing, and thus various types of nanoparticles such as carbon, polymeric, lipid-based, ceramic nanoparticles, and metal-based materials have further opened up the untapped potential in composite design [172]. In general, improved mechanical properties and bioactivity of the final binary systems have been claimed. For example, mesoporous bioactive glasses (MBGs) as carriers for the delivery of biomolecules in the physiological processes have been incorporated into biopolymer-based hydrogels to provide multifunctional biocomposites, finding promising potential in constructing tissue engineering scaffolds [173,174]. Nanotechnology utilizes bottom-up or top-down approaches to engineer nanoparticles (NPs) from single groups of atoms, molecules, or molecular aggregates, or reducing large materials, respectively, combining interdisciplinary fields of science such as chemistry, physics, biology, and engineering. In anti-cancer therapeutics, targeted nanotechnology-based drug delivery systems are advantageous over conventional treatments and allow for administration of both hydrophilic and hydrophobic substances, as well as enable improved biodistribution of anti-cancer drugs via various administration routes with enhanced therapeutic efficacy and reduced side effects [175].

A few examples of US FDA and European Medicines Agency (EMA) approved nanotechnology-based drug-delivery systems are albumin-bound paclitaxel-loaded NPs from Abraxis Bioscience, PEGylated liposomal daunorubicin from Diatos, PEGylated liposomal doxorubicin from Ortho Biotech and Schering-Plough, and liposomal doxorubicin from Elan Pharmaceuticals [21,176]. The first two COVID-19 vaccines to gain emergency-use approval by Moderna and Pfizer/BioNTech were lipid NPs carrying mRNA [177] and are regarded as a huge advance by the nanomedicine field in gaining widened public acceptance of nanomedicine-based technologies [177].

**Figure 4:**
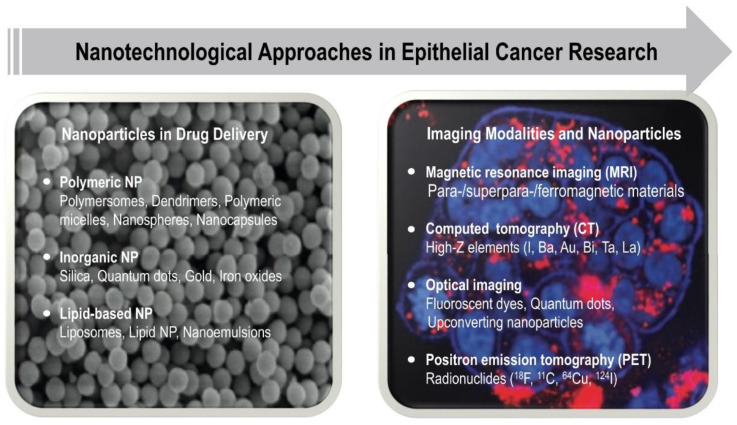
Overview of different types of nanoparticles used in drug delivery and imaging for epithelial cancer research.

After the first approval of Doxil^®^ in 1995, cancer therapeutics has remained the main “beneficiary” of nanomedicines, and thus, the extensive research in this field have yielded different types of nanoparticle systems useful for cancer therapy and diagnostics such as liposomes, polymeric, micellar, protein, viral, metallic, and lipid NPs [176,178,179] (Figure 4). Similarly, various types of mostly inorganic nanoparticles, such as (super)paramagnetic iron oxides and gold nanoparticles, are being used as magnetic resonance imaging contrast agents for intraoperative imaging in cancer diagnostics to visualize tumors. As for the biomaterials discussed above, composite or hybrid nanomaterials can be constructed that render them multifunctional, thus being able to simultaneously provide imaging activity and targeted drug delivery (so-called “theranostic agents”) [22,180,181,182,183]. Although the advancement of nanotechnological approaches for anti-cancer therapeutics have come a long way during the past two decades and today provide a new ray of hope for personalized medicines, grand challenges remain related to biocompatibility, tumor-targeting potential, chronic toxicity, biodegradability, cost-effectiveness, and so forth, all of which need to be rationally addressed in preclinical and clinical research [184,185].

### 6.1. NPs Evaluated on 2D Cell Cultures

Nanomedicine refers to a wide range of applications of nanotechnology in medicine, including drug delivery, therapeutics, imaging, diagnostics, and the construction of active implants [175]. To date, the 2D cell culture system still is routinely used as an initial tool in vitro for nanomedicine evaluation in cancer biology research for its simple and low-cost maintenance. The 2D cell culture systems always require cells with adherent ability to the plastic of culture flask or Petri dishes, on which single-cell layers could be formed for drug development testing and cell biology studies [186]. Although 2D cell culture models could support the drug safety and efficacy assessment, it is not biologically representative of the in vivo TME and tissue structure [186]. In conventional 2D models, many complex biological responses, such as receptor expression, cell signaling, cell morphogenesis, polarization, differentiation, and invasion to either differ significantly or lack completely [187]. For example, adherent cells would express enough surface receptors to recognize cell attachment motifs that are adsorbed onto the culture surface, while in tissues, multiple functions such as physical, mechanical, biochemical properties of cells are regulated by discrete interactions with ECM [188]. Standard 2D culture systems also lack the interaction between different cell types, therefore, responses to nanomedicines could be significantly altered in 2D cell cultures when compared to organotypic 3D cell culture or tissues in vivo [187].

The simplest limitation in the context of nanomedicines is the effect of gravity. Unlike drug molecules that are dissolved in the cell media, nanomedicines are macroscopic objects (materials) that may likely be affected by gravity when suspended in media. Thus, when adding NPs to cell media where the 2D cell culture lays on the bottom of the dish, sooner or later, the NPs will sink on top of the cells. This may lead to misrepresentation of the extent of cellular uptake both in terms of detection method and extent of exposure (“where else would they go”). NPs that end up on top of cells as a result of gravitational descent and thus adhere to the cell membrane may be erroneously detected as cellularly internalized in fluorescence-assisted cell sorting (FACS). First, NPs may adhere strongly to the cell membrane and not easily be washed away during the washing steps used in the FACS preparation of cells. Even if fluorescence quenching is used to eliminate the extracellular fluorescence, the protocols developed for the quenching of molecules may not prove effective for quenching of NPs. Fluorescence quenchers also exist for a very limited number of dyes (most commonly trypan blue is used for the quenching of fluorescein), while any fluorescent dye may be used to fluorescently label NPs – unless they are inherently fluorescent, which also most likely would render the quenching mechanism completely different that that of molecular dyes. Fluorophores embedded inside the NP matrix may also not be accessible for the quencher, even if one would exist. NPs sinking to the bottom of a cell culture dish also do not correctly represent the exposure of NPs in 3D. Since one of the foremost advantages of nanomedicines in cancer therapy is the ability to cross biological barriers, the physiological representation of which does not exist in 2D models (except for when the barrier is a monolayer of cells). As already outlined above, several physiological barriers exist in tumor tissue, and thus, a successful anti-cancer nanomedicine would be able to bypass these with deep tumor penetration and drug delivery as a result, all of which have to be recapitulated with the aid of 3D models.

### 6.2. NPs Evaluated on 3D Cell Cultures

3D culture models, including both 3D non-scaffold-based cell cultures and 3D scaffold-based cell cultures, have been proven to mimic certain parts of the microstructure, dynamic mechanical properties, and biochemical functionalities of whole living organs, offering an attractive application value in nanomedicine assessments [189,190]. The 3D spheroids and organoids deposit ECM constituents, such as collagen, fibronectin, tenascin, thus mimicking different epithelial cancer types in vitro. Currently, the techniques for producing a large number of spheroids at a low cost allow the application of spheroids to optimize a nanomedicine’s physicochemical properties such as size and surface charge distribution, shape, and surface chemistry to meet the criteria of the foreseen application [189,191]. Moreover, the 3D cultures present enormous potential in the screening of therapeutic efficacy in the field of chemotherapy [192,193], radiotherapy [194,195], photothermal therapy [196,197], photodynamic therapy [198,199], and gene delivery [200,201], since various available fluorescence microscopy detecting techniques, confocal laser scanning microscopy, light-sheet-based fluorescence microscopy, two-photon/multiphoton microscopy, and single (or selective) plane illumination microscopy could be used [191]. Organ-on-a-chip 3D models with microfluidics, cells/tissues/organs, stimulation, and sensor systems present a unique platform for the screening of nanomedicines in terms of hemo- and biocompatibility, toxicity, and uptake, accumulation, and targetability [199,200,201].

**Figure 5:**
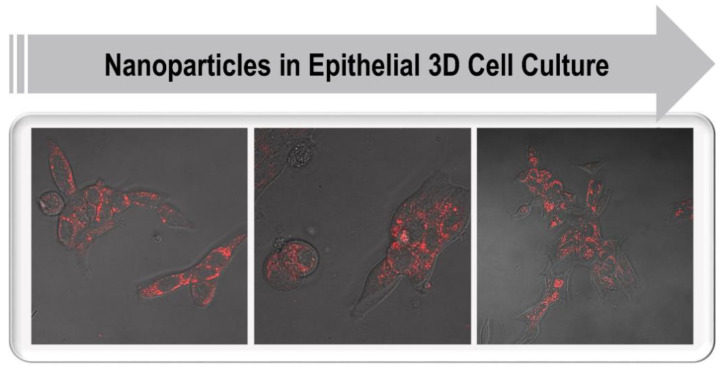
Fluorescent nanoparticle uptake in LNCaP prostate cancer cells cultured in 3D using collagen. When up-scaled, these cultures will be beneficial for high-content imaging.

In general, 3D cell culture systems provide excellent preclinical screening tools when transferring nanomedicines into clinical practice. Conversely, NPs can also constitute excellent long-term cellular labeling agents for distinguishing and tracking different cell types in 3D models or in vivo [202]. It is first necessary to test NPs in 3D cultures that mimic the TME found in tissues, allowing assessment of NP penetration across multicellular structures such as the basement membrane, and elucidating their interaction with different stromal cell types (Figure 5). In addition, it is critical to further validate the relationships between NP properties and their behavior in tissue-like environments, including natural ECMs, synthetic polymers, or composite materials. Here, a screening platform would function as a tool to select NP preparations that show the best tumor-specific delivery. Thus, the specific functionality of NP preparations could systematically be tested using a high-content screening platform based on 3D cell cultures.

### 6.3. High-Content Imaging and Nanotechnology in 3D

One advantage of high-content screening over other high-throughput screening platforms is that it provides information about functional data points together with spatial information in 3D [203]. This allows cell-based screening to progress towards more complex, 3D mono- and co-culture models. In order to have a functional high-content screening platform compatible with complex culture systems, the platform should allow simultaneous automated imaging, segmentation, and quantification of different cell compartments. This is particularly important when the role of tumor-stroma interactions is investigated [60]. The integration of high-resolution imaging will be especially useful for the evaluation and functional testing of distinct NPs. Including robotic and liquid handling equipment can also be an effective addition to the high-content screening platform when there is a need for higher throughput. Furthermore, the screening approach should allow a precise efficacy association of drug action with representative tumor morphology, such as changes in tumor cell invasion or increased epithelial differentiation. Phenotypic changes should be easily quantified by automated image analysis and provide a simplified readout for changes in tumor growth, morphology, and invasive properties.

New and better-targeted therapies are needed for epithelial cancers, in particular for the advanced and metastatic stages. It would be extremely important to develop 3D spheroid/organoid platforms for high-content drug discovery in oncology, especially with the focus on late-stage disease [204]. The main focus should be on mimicking and capturing dynamic processes involved in cancer progression and therapy resistance. The platform should recapitulate not only tumor architecture but also dynamic processes and heterogeneity, which are observed in the patient tissues, a more complete, tissue-like composition of organoids, supported by the structurally defined ECM or scaffold. At least one or the other of these components are often missing in 3D screening platforms, or the readout is not informative enough for predicting drug effects in humans. However, there are examples of successful studies that have demonstrated the practical implementation of 3D cultures and high-content screening platforms [60,205,206,207,208], even if their adoption into preclinical routine screening has been slow, partly due to technical problems. A major challenge is, for example, in retaining the resolution of the cellular details and performing simultaneous imaging and analysis of multicellular 3D structures at a larger scale.

In parallel, nanotechnology tools for drug delivery and cell labeling should be integrated into a standardized and automated platform to support high-content screening applications. With such combined solutions, it should be possible to provide novel tools for personalized medicine and chemosensitivity testing in vitro. A spectrum of nanoparticle-based imaging probes could be used to study the different cellular components of the TME, to target certain cell populations over others in 3D co-culture settings, and also for long-term labeling and monitoring of cell populations. The NPs used in the screening platform would serve as a carrier for fluorescent dyes that can be released specifically inside target cells [209,210], or the NPs are themselves fluorescently labeled to serve as an imaging probe or even both simultaneously. After the NPs have been used to optimize and characterize the 3D cultures of the screening platform, the same platform and readout (intracellular delivery of active compounds) could be utilized to gain critical information for designing novel nanoformulations for drug sensitivity tests. Only a few reports have so far shown successful development and use of NPs in high-content screenings. For example, Cutrona and Simpson developed a confocal microscopy-based screening platform that enabled quantitative morphological profiling of colorectal cancer spheroids. The platform was used to demonstrate a quantitative dissection of the penetration of synthetic NPs in 3D spheroids [211]. In the future, the complex 3D culture models in combination with quantitative imaging approaches have the potential to be adopted more often for evaluations of cytotoxic and phenotypic effects based on NP-mediated drug delivery. With such in vitro 3D high-content screening strategies, better and more biologically relevant information would be gained on how therapeutics can cross different ECM types and cell compartments in epithelial tumor tissues.

## 7. Conclusions

In epithelial cancer research, the synergy between basic research and technological advancement has provided substantial information about the cancer origin, TME, and cellular mechanisms involved in tumor progression. There is a tremendous potential for 3D in vitro models that very closely mimic the in vivo model situation and recapitulate the cellular mechanisms involved in tumor progression. The possibility to design cell models by combining patient-derived cells and biomaterials utilizing 3D bioprinting technology has provided hope and motivation when studying cancer genetics and invasion mechanisms, but also facilitating the screening of anti-cancer drugs. In anti-cancer therapeutics, the crucial role of targeted nanotechnology-based drug delivery systems has proven advantageous over conventional treatments, thus enabling the improved biodistribution of anti-cancer drugs via various administration routes with enhanced therapeutic efficacy and reduced side effects. However, the translation from bench to bedside has not been as rapid as initially hoped for, partly due to a lack of suitable platforms for nanomedicine evaluation in a relevant setting. Therefore, the greatest potential relies on using high-content imaging combined with complex 3D culture models including ECM and the generation of a platform that not only addresses the TME combined with physiologically relevant ECM but is also suitable for screening and testing of NP delivered drugs on larger scale.

Nevertheless, the chosen biomaterial has to meet several categories for TME in terms of mechanical and biochemical properties. New materials need to be developed in order to create relevant 3D platforms. The selection of material substrates and further chemical design approaches should keep those categories as prerequisites. One single material cannot meet all the required properties, and thus composites with dual- and multicomponent structures could be applied to tailor optimal systems. The advancement of bioprinting techniques enables the extended possibility to tailor materials in a customized manner and create biomimetic 3D environments. However, the design of biomaterials for ink formulation is still a challenge and thus requires active dialogues between cell biologists and material scientists.

Miniaturized and standardized high-content screening platforms for the investigation of NP behavior are still very rare. In the future, quantitative high-content imaging approaches will most likely be utilized more for the profiling of the effect of drug-loaded NPs and the evaluation of anti-tumorigenic effects, and better information on how therapeutics interact with ECM or scaffold and different cell types in tissues. The technological development enabling such in-depth studies tandem with higher throughput capacity has been exceptionally rapid during recent years, spurring high hopes of the likewise rapidly developing 3D models to be utilized to their full potential in the development of new medicines.

In summary, 3D cell models have unique advantages compared to 2D models since they mimic the TME and recapitulate the cellular and ECM crosstalk. In addition, (targeted) nanotechnology-based drug delivery systems have proven advantageous over conventional anti-cancer treatments. Therefore, it is crucial that complex 3D models combined with high-content imaging platforms also will be suitable for screening and testing of NP delivered drugs and evaluation of their anti-tumorigenic effects.

## Data Availability

Not applicable.
